# Robust Microplate-Based Methods for Culturing and *in Vivo* Phenotypic Screening of *Chlamydomonas reinhardtii*

**DOI:** 10.3389/fpls.2018.00235

**Published:** 2018-03-22

**Authors:** Timothy C. Haire, Cody Bell, Kirstin Cutshaw, Brendan Swiger, Kurt Winkelmann, Andrew G. Palmer

**Affiliations:** ^1^Department of Biological Sciences, Florida Institute of Technology, Melbourne, FL, United States; ^2^Department of Chemistry, Florida Institute of Technology, Melbourne, FL, United States

**Keywords:** *Chlamydomonas reinhardtii*, *in vivo* photosynthetic assays, *Chlamydomonas reinhardtii* viability, *Chlamydomonas reinhardtii* microplate-based culture, photosynthetic pigment analysis, high-throughput screening of Chlamydomonas, nanoparticles, *Chlamydomonas reinhardtii* toxicology

## Abstract

*Chlamydomonas reinhardtii* (Cr), a unicellular alga, is routinely utilized to study photosynthetic biochemistry, ciliary motility, and cellular reproduction. Its minimal culture requirements, unicellular morphology, and ease of transformation have made it a popular model system. Despite its relatively slow doubling time, compared with many bacteria, it is an ideal eukaryotic system for microplate-based studies utilizing either, or both, absorbance as well as fluorescence assays. Such microplate assays are powerful tools for researchers in the areas of toxicology, pharmacology, chemical genetics, biotechnology, and more. However, while microplate-based assays are valuable tools for screening biological systems, these methodologies can significantly alter the conditions in which the organisms are cultured and their subsequent physiology or morphology. Herein we describe a novel method for the microplate culture and *in vivo* phenotypic analysis of growth, viability, and photosynthetic pigments of *C. reinhardtii*. We evaluated the utility of our assay by screening silver nanoparticles for their effects on growth and viability. These methods are amenable to a wide assortment of studies and present a significant advancement in the methodologies available for research involving this model organism.

## Introduction

The unicellular alga *Chlamydomonas reinhardtii* is a model organism for investigating cellular phenomena such as photosynthesis and motility, as well as sexual and asexual reproduction (Harris, 2001; Renaut et al., 2006; Marshall, 2009; Zaffagnini et al., 2012). This photoautotroph has minimal nutritional requirements, is genetically tractable with full sequences of its chloroplast, mitochondrial, and nuclear genomes available, and has an extensive strain repository. These advantages have made this an ideal organism for the biotechnology sector, most notably in the areas of biofuel and protein production (Scranton et al., 2015). *C. reinhardtii* has also served as a proxy for both plants and humans, as a model for photosynthesis or ciliary diseases, respectively (Dent et al., 2001; Stolc et al., 2005). *C. reinhardtii*-based studies can provide essential preliminary results in less time, at reduced costs, and without the other restrictions associated with multicellular plants, animals, or eukaryotic tissue culture.

Microplate-based screening of large compound libraries, growth medias, and/or mutants is a powerful tool for drug discovery, functional genomics, toxicology, and dissecting biological processes (Dan et al., 2006; Engel et al., 2011; Serrano et al., 2015). Plate-based approaches, such as high-throughput screening, typically involve the dispensing and growth of cells at small volumes (≤200 μl) in wells of a plate that are subsequently analyzed for changes between control and test samples by one or more spectrophotometric approaches such as absorbance, fluorescence, or luminescence. Traditional flask-based approaches require larger spaces, more reagents, and greater media volumes, often 100- or 1000-fold more than microplates. As a result, microplates facilitate the rapid analysis of significant sample sizes at reduced costs and with less waste. Often they provide the only cost and/or time effective way to screen assays with 100+ possible distinct entries (Zhu et al., 2014).

Considering the potential advantages a microplate-based approach might provide, we sought to develop such an assay for screening chemical or environmental impacts on *C. reinhardtii*. Beyond the ability to evaluate the effects of multiple compounds and/or conditions simultaneously on growth, a microplate assay for *C. reinhardtii* would permit more subtle changes to be evaluated as well. For example, in both plants and algae, chlorophylls and carotenoids play integral roles in light harvesting and mediating stress responses to a variety of endogenous stimuli, including: salinity, pathogenic infections, and oxidative stress. Changes in photopigment concentrations can be effective markers of these stimuli (Peñuelas et al., 1993; Lobato et al., 2010; Havaux, 2014). Unlike plants, which typically require homogenization and extraction to quantify photopigments, liquid *C. reinhardtii* cultures could potentially be analyzed spectrophotometrically with the same plate reader used to acquire other data.

*Chlamydomonas reinhardtii* has previously been cultured in microplates (Marshall, 2009; Engel et al., 2011). However, these studies made no comparison between microplate and traditional flask cultured cells. Yet, adapting growth studies of any cell line or microorganism to microplate-based approaches comes with distinct challenges and should not be undertaken without a number of control studies. For example, when dealing with low volumes of media (<200 μl) it is important to balance sample aeration with evaporation, to ensure that volumes remain constant within test wells. Unequal evaporation across the plate is one of the primary sources of plate-based artifacts, often referred to as ‘plate effects.’ Second, the turbulent patterns that influence culture aeration, and ultimately growth, depend greatly on the type of vessel as well as fluid volume (Duetz et al., 2000). Ultimately, cells cultured in flasks and those cultured in microplates may have different doubling times, cell morphologies, etc. and care should be taken to evaluate these concerns whenever new microplate-based assays are being developed.

In addition to the challenges associated with transitioning any microorganism from flask to microplates, there are several *C. reinhardtii*-related features that may further complicate such an effort (Van Wagenen et al., 2014). Aeration and photoperiod are particularly important components in the growth of *C. reinhardtii*, as well as other photoautotrophs. Additionally, the current standard for determining *C. reinhardtii* concentrations is through manual counts of fixed cells using a hemocytometer, which is impractical for plate based assays that can easily exceed 100 individual samples (Therien et al., 2014). Optical density has been previously explored as a measure of cell concentration, but has lacked verification or correlation to actual cell concentrations, or use wavelengths overlapping photopigment absorbances (Chen, 1996; Piasecki et al., 2009; Kong et al., 2010; Alfred et al., 2012). Resolving this discrepancy would support an automated approach based on spectrophotometry to non-destructively quantify large sample sizes or limited sample volumes. *C. reinhardtii* cells range from 75 to 150 μm^3^, depending on nutrient availability, cell age, or stage of development, potentially complicating absorbance-based correlations to cell concentrations (Umen and Goodenough, 2001). Also, despite its motility, these populations are prone to settling in liquid media unless agitated complicating measurement efforts (Wakabayashi et al., 2011; Nonaka et al., 2016). Any plate based assay would therefore need to consider how to ensure proper sample mixing so the data collected between samples (wells) are comparable.

Here we present a robust microplate-based assay that permits real-time measurements of *C. reinhardtii* growth, viability, stress, and photosynthetic pigments. We have leveraged the extensive body of work for this organism to adapt existing protocols for microplate-based methods, while also adding new techniques as well. In order to evaluate the utility of our method, we investigated the toxicity of silver nanoparticles on *C. reinhardtii*. This organism has been utilized extensively as a model system for nanoparticle toxicity making it an ideal selection for assay validation (Perreault et al., 2012; Leclerc and Wilkinson, 2014; Sørensen and Baun, 2014; Navarro et al., 2015; Lei et al., 2016). Our assay successfully observed differences in both the toxicity and potentially the mode of action between silver cations and complexed nanoparticles. Our refined microplate assay expands the tool box available for this model organism.

## Materials and Methods

### General Methods

The common wild type strain CC-124, also known as 137c, was chosen for this study and obtained from the Chlamydomonas Resource Center^1^. Growth conditions varied throughout trials in terms of media, vessel type (flask or microplate), microplate shaker speed (flasks were grown at 100 rpm), and photoperiod as indicated. Cultures were maintained at room temperature (≈22°C) with an overhead fluorescent light source (250 μmol photons/m^2^/s) with Tris-Acetate-Phosphate (TAP) media or Tris minimal media (reduced nitrogen) (Gorman and Levine, 1965). Liquid cultures were inoculated from pre-grown plates, and grown for 48 h.

Aliquots from these cultures were diluted to ≈2.5 × 10^5^ cells/mL at the start of each experiment. *C. reinhardtii* was grown in either 125 mL flasks or 96 well microplates in either 1/5 (25 mL) or 2/3 (200 μL) total liquid volumes, respectively, to ensure aeration. An improved Neubauer hemocytometer and microscope (200–400× magnification) were used to measure cell concentrations manually. Flask and microplate cultures were grown for 72 h under day:night (16:8 h) and continuous photoperiods examining different microplate shaking speeds (100, 150, and 300 rpm). Each sample type had three biological replicates. Cell concentration was measured with a hemocytometer at each timepoint (0, 3, 6, 9, 12, 24, 36, 48, and 72 h) in triplicate. Growth curves were plotted from these results and were compared to the 100 rpm flask with the R statsmod compareGrowthCurves function with a threshold of *p* < 0.05. Mixing between wells as well as evaporative loss was prevented by using clear plastic plate covers that were sterilized with 70% ethanol.

### Automated Cell Concentration Estimation

The absorbance spectrum of *C. reinhardtii* cultures (TAP + 0.5% DMSO) was examined at 1 nm intervals from 300 to 800 nm using an i3 Spectramax plate reader. Cell concentrations were measured in triplicate with a hemocytometer, and OD_550_ was also recorded for each individual sample. Manual measurements were correlated to OD_550_ measurements with a third order polynomial, the raw OD_550_ values were converted through their respective polynomials to generate predicted values, and the predictions were then compared to hemocytometer measurements with regression analysis.

### Media Variance and DMSO Compatibility

Cultures were grown in either flask or microplates (100 and 300 rpm, respectively) with TAP, TAP-DMSO (0, 0.5, 1, and 2% v/v), or Tris-minimal media (low nitrogen) (Gorman and Levine, 1965). Each sample type had four biological replicates. Cell concentrations were manually measured with a hemocytometer at 0, 24, and 48 h, and then analyzed with either a Welch’s ANOVA and Dunnett’s *t*-test *post hoc* comparison at each timepoint to the flask TAP control, or an independent *t*-test at each timepoint between vessels of the same media type.

### Viability Measurements

Fluorescein diacetate (FDA λ_ex_: 493 nm, λ_em_: 523 nm) was added at a final concentration of 2.4 μM (1 μg/mL) to *C. reinhardtii* cultures (TAP + 0.5% DMSO). Samples were incubated in the dark at room temperature for 30 min before measuring fluorescence. Heat treating the samples for ≈45 min at 90°C was used to establish cell death and act as a negative control for viability (Vavilala et al., 2016). A modified version of FDA, 2′,7′-dichlorodihydrofluorescein diacetate (H_2_DCFDA), was used to measure reactive oxygen species (ROS) at a final concentration of 100 μM (48.73 μg/mL). Incubation times and measurement settings were identical to FDA.

Two Promega (Madison, WI, United States) viability kits were also evaluated – CellTox Green Cytotoxicity Assay (membrane integrity) and RealTime-Glo MT Cell Viability Assay (reducing potential). These kits were multiplexed, testing both assays simultaneously in each sample, in accordance to manufacturer instructions as an endpoint assay following heat treatment. All viability assays were performed on an i3 Spectramax plate reader. Cell concentration and percent viability were recorded, and a Welch’s ANOVA was performed for each at 48 h.

### *In Vivo* Photopigment Concentration Estimation

Photopigment concentrations were measured with the standard Lichtenthaler 80% acetone extraction method – cell cultures were pelleted through centrifugation (8 min, 10,000 × *g*), resuspended in an equal volume of 80% acetone, vortexed (30 s), centrifuged under the same conditions again, and the resultant supernatant was measured spectrophotometrically at 470, 647, 663, and 750 nm (Lichtenthaler, 1987). These wavelength measurements were blanked by subtracting A_750_ from each other value before calculating pigment concentrations using the following equations: ChlA (μg/ml) = 12.25(A_663_)–2.79(A_647_), ChlB (μg/ml) = 21.5(A_647_)–5.1(A_663_), Total carotenoid (μg/ml) = [1000(A_470_)–1.82(ChlA)–85.02(ChlB)]/198. For *in vivo* measurements, cell cultures were directly measured at 470, 650, 680, and 750 nm with A_750_ used again as a blank. The extraction values were correlated to the *in vivo* values for each pigment with third order polynomials, and regression analysis was also performed between the predicted and actual measures.

### Silver Nanoparticle Synthesis

Silver nanoparticles were synthesized by a procedure modified from those described by the California NanoSystems Institute^2^. In brief, silver nanoparticles were synthesized by heating a solution consisting of equal volumes silver nitrate (0.0047 M) and sodium citrate (0.20 M) at 80°C for 30 min.

## Results

### Microplate Growth

**FIGURE 1:**
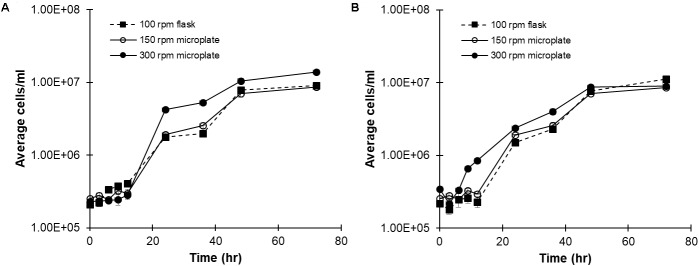
Comparing *C. reinhardtii* growth in flasks and microplate wells. CC-124 cultured with TAP medium under **(A)** 16:8 h day:night or **(B)** continuous photoperiods in either flasks or microplates at the indication shaker speeds. Cell concentration was determined at the indicated time points by hemocytometer (*N* = 3).

Cultures of *C. reinhardtii* CC-124 were grown in both flasks and microplates to determine the effect, if any, exerted by container type. Cultures were grown for 72 h with either a 16:8 h day:night cycle (**Figure 1A**) or a continuous photoperiod (**Figure 1B**). The 16:8 photoperiod reflects natural lighting conditions, while the continuous photoperiod is used to maximize growth for industrial applications. Sterile plate-seals were used to prevent evaporation. Considering the importance of proper aeration to *C. reinhardtii* growth, microplate cultures were grown at 300 rpm initially to better mimic flask aeration (100 rpm). Cell concentrations were manually measured, by hemocytometer, in triplicate at 0, 3, 6, 9, 12, 24, 36, 48, and 72 h intervals. A growth curve for each photoperiod (16:8 day:night or continuous), shaker speed (in rpm), and vessel type (flask or plate) is shown in **Figure 1A** (16:8) and B (continuous). The 300 rpm samples actually exceeded the growth of *C. reinhardtii* in flasks under both lighting conditions. Reducing the shaking speed to 150 rpm resulted in microplate cultures which mimicked growth in control flasks under similar lighting conditions (**Figures 1A,B**).

### Automated Cell Concentration Estimation

**FIGURE 2:**
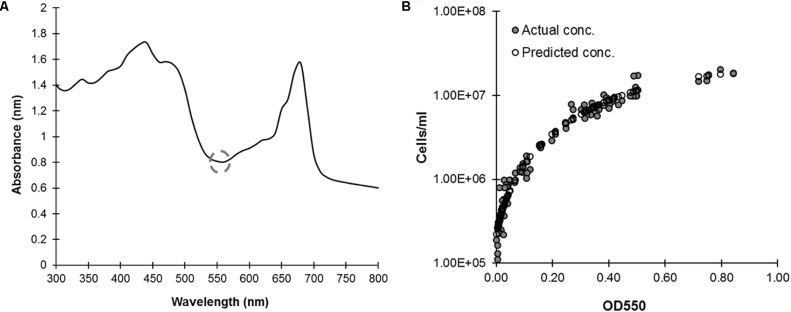
Calculating *C. reinhardtii* cell concentration based on optical density. **(A)** *In Vivo* absorbance spectrum of CC-124 cultured in liquid TAP in 96-well microplates at 150 rpm under continuous lighting for 48 h. An absorbance minimum at ≈550 nm is highlighted by the gray circle (dashed). **(B)** 48 h cultures were diluted in a series, then measured for absorbance at 550 nm and their actual cell count determined by hemocytometer (gray circles). Cell concentration is reproducibly correlated (*R*^2^ = 0.94) (*N* = 4) to optical density (OD) at 550 nm (white circles) with a third order polynomial: Cell Concentration = (216944) + (8483581^∗^(OD_550_)) + (46233132^∗^(OD_550_^2^)) + (–36516574^∗^(OD_550_^3^)).

Manually determining *C. reinhardtii* cell concentration is time consuming and subject to sampling and human errors; automating these measurements would reduce these negative factors and is necessary for making this organism viable for any sort of high-throughput, real-time assays. To correlate manual cell concentration counts to absorbance we first needed to identify an appropriate wavelength, one generally free of existing signal. The full absorbance spectrum of *C. reinhardtii* cultures in 1 nm increments noted a relatively signal free region around ≈550 nm between the chlorophyll peaks (**Figure 2A**). Using this wavelength, and a known series of culture dilutions, we developed a correlation curve to predict cell density with an *R*^2^ ≈ 0.94 (**Figure 2B**). This assay accurately predicts cell concentrations from 2.8 × 10^5^ cells/mL and higher with an average absolute value of percent deviation from known at 16.16% ± 1.38 (SE). This is an acceptable range for preliminary plate-based screens to provide leads for further testing.

### Media Variance and DMSO Compatibility

**FIGURE 3:**
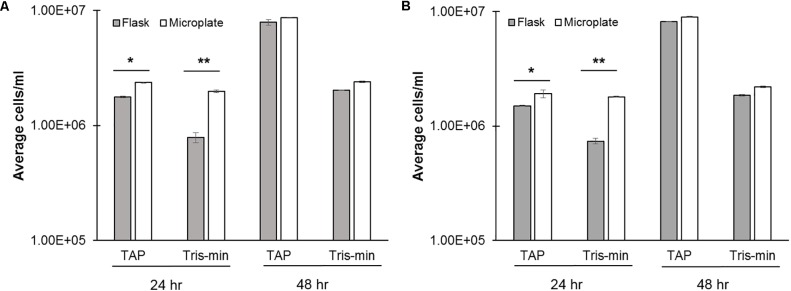
Effects of media type on *C. reinhardtii* growth. CC-124 was cultured with different medias in either a flask (gray; 100 rpm) or microplate (white; 150 rpm) under **(A)** 16:8 h day:night or **(B)** continuous light cycles. Cell concentrations were measured at 24 and 48 h (*N* = 4). A Welch’s ANOVA and Dunnett’s *t*-test were used to determine statistical significance compared to Flask TAP controls at ^∗^*p* ≤ 0.05 or ^∗∗^*p* ≤ 0.03.

Nutrient availability is an important consideration in *C. reinhardtii* growth, motility, and for biotechnology applications, such as biofuel production. TAP is a relatively, nutrient rich media for the growth of *C. reinhardtii*, so we next wanted to test growth under reduced nutrient conditions. Tris-minimal (nitrate free) media has previously been shown to reduce growth in flasks (Dean et al., 2010). Indeed, at 24 h in Tris-minimal media, flask growth was reduced by approximately 50% relative to TAP controls, while microplate growth was not significantly altered (**Figures 3A,B**). However, at 48 h both flask and microplate growth in Tris-minimal media were comparably reduced relative to TAP controls. These findings were comparable across both the continuous as well as the 16:8 day:night cycle.

**FIGURE 4:**
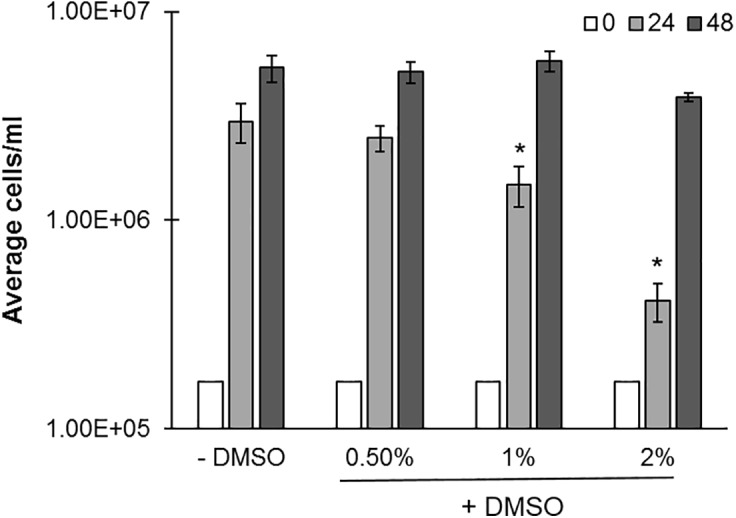
Effects of DMSO on *C. reinhardtii* growth. cc-124 cultures were grown in microplates under continuous lighting at 150 rpm over a 48 h period in the indicated concentrations of DMSO (*N* = 4). Samples were evaluated for culture growth at 24 and 48 h intervals. R statsmod curve comparison tool was used to indicate statistical significance at *p* ≤ 0.05 (indicated by ‘^∗^’) relative to untreated (–DMSO) controls.

Dimethylsulfoxide (DMSO), is a common carrier solvent for small molecule screening in biological assays, and has been previously evaluated in *C. reinhardtii* (Alfred et al., 2012). However, DMSO becomes toxic to cells at higher concentrations, and a dosing range which would not alter the growth of Cr needed to be established (Alfred et al., 2012). Microplate cultures were therefore evaluated for growth at 0, 0.5, 1, and 2% DMSO (v/v) (**Figure 4**). Increasing DMSO concentrations lead to a decrease in cell concentrations, most notably at 24 h (50–80%), and to a lesser extent at 48 h. 0.5% DMSO did not appreciably change compared to the untreated control. We conclude that 0.5% DMSO and not 2% is an acceptable concentration range for studies with small molecules at 24 and 48 h timeframes as this ensures a similar growth ‘trajectory.’

### Viability Measures

**FIGURE 5:**
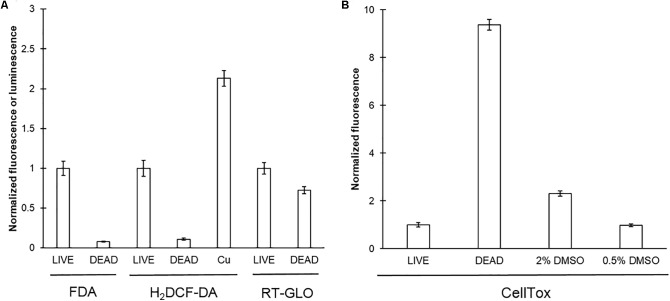
Evaluating *C. reinhardtii* viability assays. CC-124 cultures were grown under continuous lighting in liquid TAP for 48 h with any indicated supplements (DMSO or 200 μM CuSO_4_) at 150 rpm. Samples were then either directly used in viability assays (LIVE) or heat treated (DEAD) for ≈45 min at 90°C. Results were normalized to LIVE controls **(A)** FDA (*N* = 3), H_2_DCFDA (*N* = 8), and RT-GLO (*N* = 5). **(B)** CellTox (*N* = 5).

While growth is an easy measure of toxicity, it provides little insight into stress related effects which may appear at lower concentrations. Numerous viability assays for unicellular organisms are available, but must be evaluated on a case by case basis. For example, the hydroxyproline rich cell wall of *C. reinhardtii* may act as a barrier to several of the common cell viability kits used with microplate-based systems (Azencott et al., 2007). FDA, a fluorescence-based viability indicator used previously in *C. reinhardtii* was chosen as a standard for comparison with other viability markers (Amano et al., 2003). Neutral FDA is able to diffuse across cell membranes and is subsequently cleaved by cytoplasmic esterases to yield anionic fluorescein (λ_ex_ = 475, λ_em_ = 535) which is typically restricted to the cell. As shown in **Figure 5**, significant fluorescence was observed in control wells with only minimal background fluorescence, while heat treated samples showed <5% of the fluorescence of the controls, consistent with the loss of esterase activity.

In order to expand the toolbox available for viability assays we included three additional approaches to assessing cell viability and/or stress: H_2_DCF-DA (2′,7′-dichlorodihydrofluorescein diacetate), as well as Promega’s CellTox Green Cytotoxicity and RealTime-Glo MT Cell Viability assays. Like FDA, H_2_DCF-DA diffuses across the membrane and reacts with cellular esterases, the subsequent H_2_DCF is then subject to oxidation by ROS to yield fluorescent DCF (2′,7′-dichlorofluorescein). As shown in **Figure 5A**, viable cells had a positive fluorescent signal, while heat-killed cells showed substantially less fluorescence (<5% of treated controls), consistent with these cells being dead, and therefore unable to engage in esterase activity. The addition of 200 μM CuSO_4_, a known stimulator of ROS production, served as a positive control for the assay (Stoiber et al., 2013).

The RealTime-Glo assay measures the reducing potential of cells through a luminescent substrate. Conversely, the CellTox Green Cytotoxicity assay measures viability by using a membrane impermeable DNA binding indicator. Increased fluorescence in this assay arises from the fluorophore intercalating with DNA which can only occur when the plasma membrane integrity is compromised, a sign of cell stress or potential viability loss. Both of these assays were viable in *C. reinhardtii*, showing increased (CellTox) or decreased (RealTime-Glo) signals, respectively, in heat treated samples relative to untreated controls (**Figures 5A,B**). We hypothesized that the reduced growth observed in 2% DMSO may well be reflected in stress/viability assays, specifically cell permeability given this chemical’s use as a carrier solvent often for lipophilic substances. Indeed 2% DMSO treatments resulted in an increase in the CellTox Green fluorescence, consistent with this hypothesis but well below the heat treated threshold (**Figure 5B**). 0.5% DMSO, however, had no apparent effect on membrane permeability by this method, consistent with this concentration being less disruptive.

### *In Vivo* Photopigment Quantification

**FIGURE 6:**
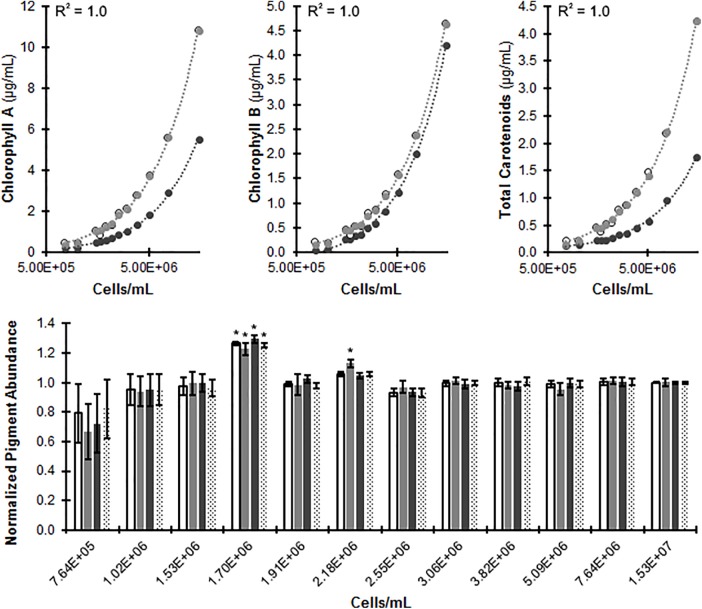
Measuring *C. reinhardtii* photopigment concentrations *in vivo*. CC-124 was cultured with liquid TAP medium in microplates at 150 rpm under continuous lighting for 48 h. (Top) Photopigment measurements of uncorrected *in vivo* values (dark circles), predicted *in vivo* values (gray; equations used for predictions indicated for each pigment) and extraction values (open circles). An *R*^2^ correlation (*R*^2^ ≈ 0.99 for Chlorophyll A, B, and carotenoids) was established through regression analysis between actual and predicted values (Top). (Bottom) Predicted *in vivo* photopigment measures were normalized to extraction values (predicted/extraction) for Chlorophyll A (white), Chlorophyll B (light gray), total carotenoids (dark gray), and total chlorophyll (dotted) (*N* = 8). A Welch’s ANOVA was used to determine statistically significant results between actual and predicted at a threshold of *p* ≤ 0.05 (Bottom) indicated by ‘^∗^’.

The photopigments, chlorophylls and carotenoids, provide valuable insight into photosynthetic potential, metabolic flux, and cell stress (Lichtenthaler and Rinderle, 1988; Boyle and Morgan, 2009; da Silva, 2016). Our plate based assay has the potential to measure relative photopigment concentrations in a non-destructive *in vivo* manner. Such non-destructive approaches would be beneficial for probing environmental, chemical, or biological effectors of *C. reinhardtii*. To develop a non-destructive assay for measuring photopigment concentrations we began by evaluating changes in the *in vitro* and *in vivo* absorbance maxima for each pigment. An absorbance spectrum of intact cells indicated a red (right) shift of the chlorophyll a (A_663_→A_680_) and b (A_647_→A_650_) maximas *in vivo*, relative to extracted pigments in 80% acetone (Lichtenthaler, 1987). Such bathochromic shifts are well documented in the literature and are generally accepted to arise from differences in the chemical environments of the pigments between *in vivo* and cell lysates (Morosinotto et al., 2003; Wang et al., 2008). These red shifted values (680 and 650 nm, respectively) were used in place of the customary *in vitro* (663 and 647 nm, respectively) values to generate representative pigment amounts (**Figure 6**). No significant red-shift was observed for carotenoids which were visible at ≈470 nm.

Representative chlorophyll a, b, and total carotenoid values were correlated to known values, and the predicted values corresponded to the actual with *R*^2^ values of 0.999 for each. Predicted pigment abundance was normalized to extraction values, and samples above 2.18 × 10^6^ cells/mL closely follow known values, while below this threshold the equation loses predictive power. We thus concluded that this *in vivo* assay produces accurate predictions of photopigment abundance with a lower limit of quantification at cell concentrations of 2.5 × 10^6^ cells/mL, and an average absolute value of percent deviation from known at 2.52% ± 0.68 (SE).

### Evaluating Nanoparticle Toxicity Through a Microplate Based Assay

Having completed the development of our plate assay, we wanted to validate its utility through a toxicity screen. Specifically, we wanted to distinguish differences in the toxicity of free silver cations and silver nanoparticles. Nanoscale materials possess unique properties due to their small size, large surface area-to-volume ratio, and quantum effects. As a result nanoparticles may be more or less toxic than an uncomplexed (free) version of the same element and distinguishing between these relative toxicities is important. Engineered nanoparticles are already found in over 2000 consumer goods and this number is to expand significantly in the next decade. It is therefore important to develop an affordable assay for screening nanoparticle toxicity and our *C. reinhardtii* screen is ideal for this purpose. Furthermore, this organism has previously been utilized for analyzing the toxicity of metal nanoparticles making this an excellent reference study for this purpose (Leclerc and Wilkinson, 2014; Navarro et al., 2015).

**FIGURE 7:**
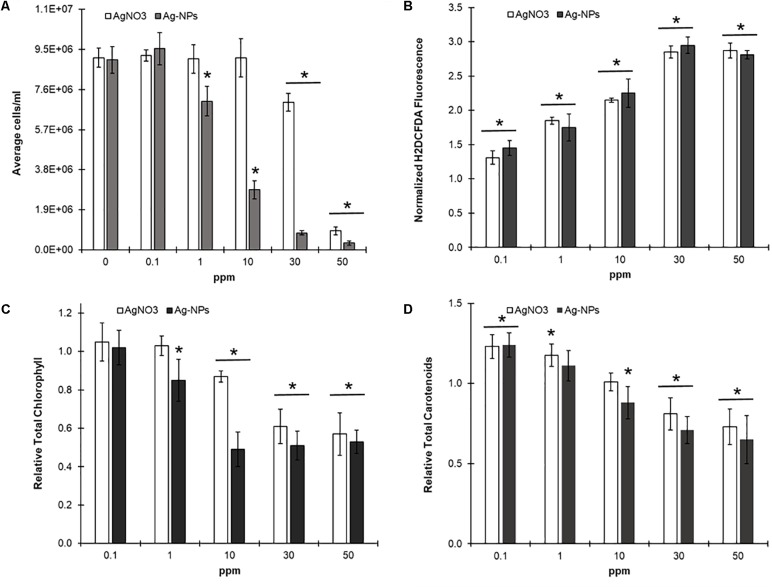
Comparing silver cation and silver nanoparticle toxicity in *C. reinhardtii* –*C. reinhardtii* cultures were grown in microplate wells in liquid TAP and the indicated concentration of either ‘free’ silver (AgNO_3_) or silver nanoparticles (Ag-NPs) for 48 h (*N* = 5) under continuous lighting at 150 rpm. Samples were then evaluated for effects on growth **(A)**, total chlorophyll **(C)**, and total carotenoids **(D)** relative to untreated controls through a multi-wavelength absorbance assay. H_2_DCF-DA was then added to evaluate for changes in ROS production as described above. **(B)** Statistically different results (*p* < 0.03) relative to untreated controls indicated by ‘^∗^’.

In order to evaluate and compare toxicities, we co-incubated *C. reinhardtii* cultures with either free silver cations or synthesized silver nanoparticles (≈40 nm) for 48 h at four different concentrations (0.1, 1, 10, and 50 ppm). After 2 days of growth we evaluated all our samples for changes in growth, photopigment, and ROS production (H_2_DCFDA) relative to untreated controls. Silver nanoparticles were noticeably more toxic than free silver, inhibiting growth by 25% at even 1 ppm (**Figure 7**). Both treatments increased ROS and carotenoid production at concentrations as low as 0.1 ppm, before any negative effects on growth were observed. However, this was followed by a significant decline in overall photopigment production as toxicant concentration increased. Despite these similarities some differences in mechanism appear to exist, as 50 ppm of silver nanoparticles clearly disrupted cell membrane integrity with the CellTox assay (sixfold increase in fluorescence, ≈3000 RFUs vs. 18000 RFUs, untreated vs. treated) while ‘free’ silver had no significant effect on fluorescence relative to untreated controls.

## Discussion

Culture and analysis of biological systems in microplates facilitates and accelerates data acquisition while drastically reducing costs associated with expensive reagents. While *C. reinhardtii* is a widely adopted unicellular eukaryotic model system, only a few examples exist in the literature of microplate cultured Cr, and these studies failed to present data to validate the use of those methods (Marshall, 2009; Engel et al., 2011). However, other species of microalgae have been adapted to microplate based culturing and high-throughput methodologies, supporting development of a similar assay for Cr (Held, 2011; Van Wagenen et al., 2014).

By evaluating growth across different vessels, media types, and concentrations of a common carrier solvent (DMSO) we have developed and validated microplate methods for Cr which approximate flask growth. Our method also allows us determine cell density based on absorbance rather than hemocytometer which is crucial for most microplate-based screens. We also confirmed the utility of FDA and membrane integrity assays (Amano et al., 2003; Sato et al., 2004) for microplate based studies, while expanding the available ‘toolbox’ of viability assays to include ROS production and cell redox potential, both of which are well-established indicators of stress. One important observation from our growth and viability assays is that higher (1–2%) concentrations of DMSO should be avoided in screening assays as their effects on membrane stability and growth likely skew results.

Quantifying photopigments is traditionally a terminal process, whereby samples are extracted in solvents and measured spectrophotometrically (Lichtenthaler, 1987). However, photopigments such as carotenoids are especially good indicators of cell stress and a microplate method to analyze this in a non-destructive fashion would be especially useful (Peñuelas et al., 1993; Lobato et al., 2010; Havaux, 2014; da Silva, 2016). Here we have established a method to determine both chlorophyll as well as carotenoid concentrations directly through absorbance measurements based on corrections due to the bathochromic (red) shift and an extraction normalization. To our knowledge no absorption spectroscopy based methodology previously existed for determining photopigment concentrations of Cr *in vivo*.

Finally, we validated our completed assay by testing for the effects of silver nanoparticles on Cr growth and viability as compared to treatments with silver nitrate. Based on these studies it is apparent that silver nanoparticles have negative impacts on growth at an order of magnitude lower concentration than free silver. This substantiates concerns that the presence of nanoparticle complexed metals may have great impacts on individual organisms, as well as communities. This is especially concerning when one considers the potential impact of destabilizing the population of photoautotrophs (producers) which form the foundation of most ecosystems.

Both free silver and nanoparticles induced increases in both ROS and carotenoid production ahead of negative effects on growth. These findings confirm our assay’s ability to observe sub-lethal stress effects beyond reductions in growth, increasing the utility of *C. reinhardtii* as a model organism in microplate-based assays. The initial increase in carotenoid production is consistent with an attempt to scavenge the elevated ROS that begins to accumulate at even 0.1 ppm of either free silver or silver nanoparticles. However, as ROS levels continue to accumulate this system likely becomes overwhelmed. While this suggests certain common modes of action between these two different species of silver, it should be noted that only silver nanoparticles showed a significant disruption in the cell membrane in response to treatments. We propose that this additional effect on membrane integrity is the source of the increased toxicity of silver nanoparticles, relative to free silver. This disruption in membrane integrity may also facilitate the uptake of other potentially toxic species amplifying the impact on photoautotroph populations. Ongoing studies are attempting to resolve more about the mechanisms associated with silver nanoparticle toxicity. We note that our nanoparticle study was conducted with less than 5% of the amount of media, nanoparticles, and reagent waste that would have been required for a flask-based study. In addition to nanoparticles, on-going studies in our lab are exploiting this assay to measure the effects of a variety of anthropogenic toxicants as well as compounds derived from a variety of microorganisms.

## Author Contributions

TH conducted most of the data analysis as well as the bulk of the viability, minimal media, and photosynthesis assays. CB performed the flask versus microplate comparisons as well as building the growth curve for absorbance studies. KC prepared the silver nanoparticle toxicity study and assisted in data analysis and editing. BS synthesized the nanoparticles. KW oversaw the nanoparticle synthesis. AP supervised all research, provided equipment, and oversaw the method development.

## Conflict of Interest Statement

The authors declare that the research was conducted in the absence of any commercial or financial relationships that could be construed as a potential conflict of interest.

## Footnotes

1 http://www.chlamycollection.org/

2 http://cnsi.ctrl.ucla.edu/nanoscience/pages/biotoxicity
